# How to Manage Diversity and Enhance Team Performance: Evidence from Online Doctor Teams in China

**DOI:** 10.3390/ijerph17010048

**Published:** 2019-12-19

**Authors:** Xuan Liu, Meimei Chen, Jia Li, Ling Ma

**Affiliations:** School of Business, East China University of Science and Technology, Shanghai 200237, China; xuanliu@ecust.edu.cn (X.L.); y30181305@mail.ecust.edu.cn (M.C.); maling@ecust.edu.cn (L.M.)

**Keywords:** team diversity, team performance, doctor teams, leader reputation, medical collaboration

## Abstract

(1) Background: Traditional one-to-one online consultations with doctors often fail to provide timely and accurate treatment plans; consequently, creating cross-hospital and cross-regional teams has become a new pattern for doctors aiming to offer Internet medical services. Because the online doctor team is a new virtual organizational model, it remains to be explained and investigated. (2) Methods: Combining the information processing view and the social categorization view, this study takes the perspective of team diversity and empirically investigates the effect of team diversity on team performance. We consider four kinds of team diversity, including status capital diversity, decision capital diversity, online reputation diversity, and professional knowledge diversity, and we investigate how team composition from the diversity perspective affects online doctor team performance and how leader reputation moderates the effect of team diversity on team performance. We use secondary data from a leading online medical consultation platform in China (Good Doctor), and our research data include 1568 teams with a total of 5481 doctors. (3) Results: The results show that status capital diversity and decision capital diversity negatively affect team performance; diversity in terms of online reputation and professional knowledge positively affect team performance; and leader reputation moderates the impact of status capital diversity and online reputation on team performance. (4) Conclusions: Our study offers management suggestions on how to form a high-performance doctor team and provides advice for the future development of online doctor teams.

## 1. Introduction

The rapid development of the Internet has greatly facilitated patients’ acquisition of medical knowledge, and the sharing of medical information on the Internet has become an inevitable trend [1]. According to the “2018 Internet Development Report of China”, as of December 2017, China’s Internet medical users increased to 253 million, an annual increase of 29.7%, accounting for 32.8% of all Internet users. Internet medical services, such as online appointments and online consultations, are the most commonly used services, and difficulties in registration and expensive medical treatment have consistently presented the most worrisome problems for patients. To solve these problems, many hospitals have undergone reforms and established well-known doctor teams.

In most developed countries or regions worldwide, the preferred method for doctors to provide freelance medical services is via a medical group [2]. Ninety percent of medical services in the United States are provided by groups of no more than 20 doctors [3]. In addition to offline medical groups, the Internet has enabled the rise of virtual doctor teams. In 2016, medical experts from several well-known hospitals in China led their teams to an online medical platform, creating a new era in online doctor teams. The online doctor team is mainly based on Internet medical consultation platforms, with a well-known expert acting as the team leader and other doctors participating as team members. The main purpose of the online doctor team is to connect patients, doctors, and hospitals through the Internet and promote the efficient sharing of medical information between the three entities. Because the online doctor team is a new virtual organizational mode, it remains to be explained and investigated.

The impact of the diversity of team members on team performance has always been a topic of great interest to scholars. The impact of the diversity of different dimensions on team performance is uncertain depending on the mediation and moderation variables [4,5]. Previous studies have focused on companies, and there has been little research on the diversity of doctor team performance. The tasks faced by the doctor team are far from those faced by the corporate team, and the mechanism of diversity will be somewhat different. This paper empirically tests how four different types of diversity affect doctor team performance under the influence of a leader’s reputation through second-hand data, which broadens the application scope of diversity theory and provides practical guidance for the formation of an efficient doctor team.

## 2. Literature Review

### 2.1. Doctor Teams and Medical Collaboration

Collaboration typically involves two or more individuals, teams, or organizations who work together to solve problems by sharing resources and skills. In this process, individuals communicate with each other to coordinate tasks [6]. Online collaboration refers to an interdependent group of individuals who collaborate across space, time, and geographic boundaries, using the Internet as a medium of communication [7,8]. Today, an increasing number of teams rely on computer-supported cooperative work to exchange ideas across regions and coordinate different tasks [9,10]. The concept of computer-supported cooperative work (CSCW) was first used to describe how computer systems support human collaborative activities [11]. The design and implementation of large-scale software systems is often a complex and costly task with a high failure rate. CSCW researchers believe that the analysis of collaborative work environments and social interactions are critical to a successful system [12]. The online consulting platform, as a third-party platform, provides a communication platform for doctors and patients and provides a collaborative platform for doctors where methods of communication include picture, text, phone, and video. Hospitals and doctors can obtain technical support by joining an online consulting platform without bearing the risk of a system failure. After the online consulting platform has formed a scale advantage, the cost becomes lower and lower and the value becomes higher and higher.

Most studies have shown that the doctor team can significantly improve patients’ symptoms [13], reduce medical risk [14], increase cure rates [15,16], and increase the sense of patients’ satisfaction or happiness [17]. Merien et al. found that a doctor team’s diagnosis can significantly reduce the rate of misdiagnosis [18]. In addition, Stephens et al. also demonstrated that a doctor team’s diagnosis increased the patients’ chemotherapy success rate from 7% to 23% and survival time from 3.2 months to 6.6 months [13]. Doctor teams serve patients with complex conditions in a multidisciplinary and coordinated manner, reducing patient hospitalization, readmission, and emergency visits [19]. Team members gain access to information and resources by building social networks that not only contain the network of relationships existing in the team but also include social capital [20]. Doctors joining the team can not only accumulate more social capital but also increase platform visibility. An online doctor team is formed by identifying a leading doctor who is usually a well-known expert in a field, and team members are then recruited online or offline by the leading doctor. Convenience and the professional skills of the doctor are the factors most often considered by the patient [21,22]. Doctor teams can provide patients with more professional and timely services. The effectiveness of a team consultation depends on a number of factors, such as team structure and composition, as well as the expertise of team members [23]. Ritholz et al. believe that maintaining good team communication and institutional factors (such as the doctor’s visit schedule and consistent team membership) play important roles in the effectiveness of a doctor’s team [24].

### 2.2. Diversity and Team Performance

Previous studies have investigated two kinds of factors influencing team performance: Team-level factors and individual-level factors [25]. At the individual level, there are a great number of studies that have considered the impact of members’ demographic characteristics [26,27] (such as race, age, gender, educational background, etc.) on team performance. There are also previous studies that have emphasized specific leaders and found that leadership characteristics affect team performance through cognition-based trust [28]. Moreover, a democratic leadership style and the leader’s position have a positive influence on team learning and subsequently influence the performance of teams [29]. Another trend of research focuses on how the diversity of a team affects team performance [27].

Team diversity is often defined as distributional differences among members of a team with respect to a common attribute [30] and may affect team performance through the process of team communication [31]. Van Knippenberg et al. summarized three review papers on the diversity–performance relationship [27]. In their review paper, Williams and O’Reilly outlined the characteristics of team diversity studies in the first 40 years and combined social classification theory with information decision theory [32]. The other two review papers focused on studies about the moderation of the diversity-performance relationship [4,5]. Many studies suggest that diversity affects team performance and member satisfaction through two mechanisms: The information processing view and the social categorization view [33,34]. The effects of diversity often depend on the interaction of the two mechanisms [31,35] and create inconsistent results. Previous research suggests that team members with a variety of skills may improve the performance of the entire team more than members with only one skill [36]. Bell et al. found that teams composed of members with different functional backgrounds should have broader perspectives and knowledge to draw on [26]. However, team diversity can sometimes lead to unfavorable group dynamics, such as high communication costs and increased conflicts [4]. In the early stages of team formation, team diversity may have a negative impact on the psychological safety and team satisfaction of team members [37]; team members with greater similarities will have greater cohesiveness because differences in team members’ easy-to-view attributes (such as race, ethnicity, language, gender, and age) are more likely to lead members to discriminate in who they interact with.

To solve the inconsistencies in the conclusions of diversity and team performance research, Van Knippenberg et al. proposed the categorization-elaboration model (CEM), which reconceptualizes the information processing view and the social categorization view, including the mediator and moderator variables. Based on the view of information processing, variables including task requirements, task motivation, and task ability moderate the relationship between diversity and team performance in the CEM model. Based on the view of social categorization, the interaction of comparative fit, normative fit, and cognitive accessibility results in social categorization, which has a negative impact on the elaboration of task-relevant information [33]. The CEM model is well supported by much empirical evidence [38,39,40]. Subsequently, more and more studies moved away from simple main effect approaches and started shifting to moderation and mediation studies [4,41]. It was justified to focus on the moderators of team diversity effects but required more integrative efforts [27].

### 2.3. Research Gap

To date, many areas have benefited from online teamwork, such as online stock trading, online shopping, and open source communities. However, the healthcare industry has adopted Internet services more slowly [42], so the pattern of online medical collaboration remains unexamined. The online doctor team inherits the model of the traditional doctor team but uses the Internet as a medium to organize the team, implement health management for patients, and enhance cross-hospital and interregional cooperation. With regard to online doctor teams as emerging virtual teams, few previous studies have studied diversity in the online doctor team, and research on the diversity of online doctor teams has not received sufficient attention.

## 3. Development of Hypotheses

To investigate the relationship between the diversity of online doctor teams and team performance, this study combines the social categorization view and information processing view. The social categorization view suggests that homogenous groups should outperform heterogeneous groups because people use silent social attributes as cues to classify themselves and others into different social categories, and working with people who are similar would improve people’s job satisfaction [26,43]. Conversely, the information processing view sheds light on the fact that heterogeneous groups should outperform homogenous groups because the former have access to a wider range of knowledge, skills, abilities, and opinions, and are thus able to generate new and unique information for better decision-making and creative solutions related to the task.

### 3.1. Diversity and Team Performance: The Social Categorization View

The social categorization view argues that team members with similar demographic attributes may be more attracted to and more likely to cooperate with each other compared to those with differing demographic attributes [26]. In addition, social categorization between team members forms an in-group and out-group distinction: People consider others who are similar to themselves in-group members, while they classify those who differ from themselves as outgroup-members. Heterogeneous groups will result in intergroup discrimination and will reduce team cohesion and increase intergroup conflict [43]. As a result, it is not surprising that studies based on social categorization generally postulate negative effects of diversity on team performance.

Online healthcare consultation between doctors and patients is a series of two-way dynamic interactions. On the online healthcare consultation platform, doctors can share medical information and provide consultations to support patients in disease prevention, diagnosis, recurrence management, and advice for self-management, and their behavior also affects patients’ trust, overall satisfaction, and desire to continue consulting with the doctor and using online counseling [44]. Meanwhile, patients can decide whether to initiate, continue, or stop communicating with the doctor, and they also evaluate the doctor’s service quality after the consultation. Generally, doctors’ participation in online health consultations is a social exchange behavior. Professional capital is a special, rare, enduring, and valuable type of capital associated with social professionals because it involves power advantages and professional commitment [45]. In the social communication between doctors and patients, the exchange resources of doctors are their professional capital, and reflect their status in the social structure and their decision-making behavior [46].

The professional capital of doctors can be classified as status capital and decisional capital [47]. Status capital represents doctors’ personal and social advantages in a social structure. It is a type of structural power that is officially certified [48] and not related to the doctor’s online behavior. For online health consultations, the status capital of a doctor is that doctor’s social level. Doctors who provide online health consultation services may have different clinical titles, such as professors or associate professors, or different positions, such as chief physicians or deputy chief physicians. Doctors with higher clinical titles usually receive greater priority and privileges. Decisional capital is considered decision-making behavior that is driven by the ability and willingness to make correct judgments [49]. In contrast to status capital, a doctor’s decisional capital cannot be recognized without dynamic interactions with patients and can be transferred into the exchange behaviors in online consultation [47]. To improve decisional capital, doctors who provide online consultation services need to signal the patients (such as providing more consulting services and posting more online articles), thereby improving the degree of trust and leading the patients to accept online consultation services.

Online reputation refers to a mechanism that manages and collects experiences and evaluations shared by participants on the Internet platform [50]. Online reputation (also known as word of mouth) can reflect a doctor’s service quality to a certain extent [51] and can help consumers offset the problem caused by partial information asymmetry [52]. For online healthcare consultation, reputation is mainly achieved by an evaluation feedback system. Various types of user feedback, such as ratings, reviews, likes, virtual flowers, and gifts, reflect users’ perception of the doctor’s service quality after receiving their consultation services. The more satisfied users are with the quality of service, the more they tend to give positive feedback, such as higher ratings, more positive reviews, more likes, more flower, or more gifts.

These three attributes of doctors are easy to view on the online consultation platform. Different doctors in a team have different attributes. Therefore, we propose the following hypotheses based on the social categorization view:

**Hypothesis** **1** **(H1).** *There will be a negative relationship between status capital diversity and team performance*.

**Hypothesis** **2** **(H2).** *There will be a negative relationship between decisional capital diversity and team performance*.

**Hypothesis** **3** **(H3).** There will be a negative relationship between online reputation diversity and team performance.

### 3.2. Diversity and Team Performance: The Information Processing View

The information processing perspective suggests that groups manage external uncertainties through an information processing system [53]. Many researchers have suggested that information processing within a team can help members overcome their information sampling bias and make better decisions, thereby enhancing performance [54]. For example, Postmes et al. show that when a criticality norm is introduced into a group, unshared information is considered more fairly, and the group can make high-quality decisions more frequently [55]. Therefore, research based on the information processing view generally posits positive effects from team diversity on performance.

Unlike highly visible attributes such as status capital, the doctors’ professional knowledge belongs to the information attribute directly related to their work, which represents the organization’s potential “information pool” and “skill pool”. At the same time, the communication between team members with different professional knowledge will promote the transfer of information to effectively complete work, enhance creativity and innovation, and thus improve organizational performance [56]. For medical collaboration, the cooperation between different departments is helpful to avoid medical error, improve patient flow and promote case sharing [57,58].

Meanwhile, members of online doctor teams interact primarily via the Internet, and these virtual teams are more prone to information processing failures such as sharing and updating team knowledge [59]. McLeod suggests that “vigilant information processing” (an as in-depth discussion of system information processing and alternative solutions) can improve the quality of decision-making for virtual teams [60]. Therefore, we expect that:

**Hypothesis** **4** **(H4).** *There will be a positive relationship between professional knowledge diversity and team performance.*

### 3.3. Leader Reputation as a Moderator

Teams should learn to work together to ensure that resources brought into the team are fully utilized to achieve collective goals, and leaders play an important role in facilitating this process [61]. The source of this leadership impact may be formal, such as a management position or leadership style in the team, or it may be informal, that is, leadership outside the team structure, such as leader reputation. Many studies have examined the moderating effect of team leaders’ individual-level attributes (e.g., their leadership style or experience) on the relationship between diversity and team performance [38,53,62].

Because diversity may lead to coordination costs and cooperation problems (i.e., the aforementioned issues of discrimination and team conflict) that must be managed, leveraging team diversity for greater team performance will often require a greater investment by the team leader [63]. In our research, leader reputation is defined as the online reputation of the leading expert of an online doctor team. Online reputation is a signal that reflects a doctor’s social returns earned from patients [47]. Studies have shown that sellers with a better online reputation in the evaluation feedback system will have a higher performance rating [64]. Reputation is also a reflection of perceived trustworthiness as evaluated by patients [44]. High levels of leader facilitation attenuate the relationship between tenure diversity and conflict [65]. Therefore, we believe that a doctor with a better online reputation will generate a higher performance rating in the online health consultation market by facilitating attenuated conflict between diversity team members.

In our research platform, more than 90% of the teams are named after the team-leading experts. Therefore, the moderating role of leader reputation on team performance cannot be ignored. Most patients may choose experts with a stronger reputation for online medical consultation, so leadership reputation has a positive effect on team performance. Thus, the differences in online reputation may affect the ability and motivation of team leaders to leverage team diversity for greater team performance. Thus, to advance the diversity and team performance relationship, we hypothesize a moderating role for leader reputation as follows:

**Hypothesis** **5a** **(H5a).** *Leader reputation positively moderates the relationship between status capital diversity and team performance.*

**Hypothesis** **5b** **(H5b).** *Leader reputation positively moderates the relationship between decisional capital diversity and team performance.*

**Hypothesis** **5c** **(H5c).** *Leader reputation positively moderates the relationship between online reputation diversity and team performance.*

**Hypothesis** **5d** **(H5d).** *Leader reputation positively moderates the relationship between professional knowledge diversity and team performance.*

**Figure 1:**
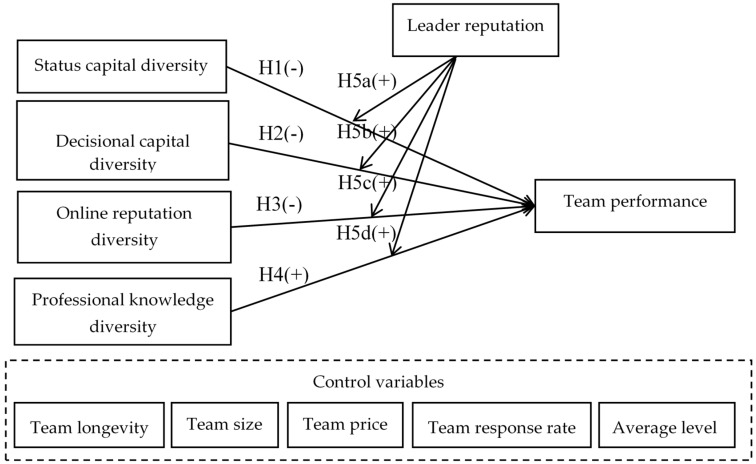
Conceptual model.

In sum, our conceptual model is represented in Figure 1.

## 4. Methodology

### 4.1. Sample and Data Collection

We test these hypotheses using a unique dataset of all the doctor teams on the online medical consultation platform, the Good Doctor (www.haodf.com). All the data are publicly available and do not involve privacy or interest disputes. The public data were crawled using network spiders on January 12, 2019.

Founded in 2006, the Good Doctor is China’s leading Internet healthcare service platform. The Good Doctor has the most authoritative and high-quality doctors in the country. On this platform, doctors can provide services for patients with different conditions; they can also form virtual doctor teams and provide services as a team. The Good Doctor is one of the most popular online medical consultation platforms in China. We collected both team-level data and doctor-level data. The team-level data included the date the teams were established, information on the team leaders, the team specialties, the team prices, the team response rates, and the number of team consultations. The doctor-level data consisted of the doctors’ personal information (e.g., hospital, department, the number of consultations, the number of letters of thanks and virtual gifts received, medical articles, and popularity). Data were collected for all teams established before January 12, 2019; after eliminating the records with missing values, there were ultimately 1568 teams with a total of 5481 doctors. The original data is linked as additional resources (data available by request from author emails). Table 1 further describes the original data and how we use those variables.

### 4.2. Measures

#### 4.2.1. Dependent Variable

Team performance (Ln). The dependent variable in this study is the team performance of online doctor teams. In our target online consultation platform, we measured this dependent variable by the natural log of the number of team consultations in line with a previous study [47]. There are different ways to measure team performance, including quantity performance and quality performance. As for quality performance, user feedback for the team could denote the service quality of the online doctor team and provide an important facet for team performance, but “joining a virtual team” is a relatively new IT artifact in the Good Doctor website, and until now the platform has not yet provide feedback function for patients to evaluate the service quality of the online team. Thus, in this study we measured team performance solely by number of team consultations. The number of team consultations was a typical variable used in previous studies to represent quantity performance of a team. 

#### 4.2.2. Independent Variables

Status capital diversity. The status capital of team members was measured by individual and social advantages. Referring to the study of Guo et al. [47], we used a doctor’s clinical title, hospital, and city to reflect status capital because the clinical title is assessed in terms of the level of technology, and a better hospital and city location indicates that colleagues or the surrounding areas hold or contain good resources that enable doctors to obtain better social advantages. According to Guo et al. [47], we first turned a doctor’s clinical title, hospital, and city into ordinal variables (title level, hospital level, and city level). Because these three variables may have different ranges, we first normalized them and then averaged them to compute the status capital. Generally, status capital suggests who and where the doctor is. In line with Harrison and Klein’s study, we computed status capital diversity using the variation coefficient of the status capital of all team members [30]. The higher the coefficient is, the more distributed the team members are, and thus the more diverse a team is. If we denote each member’s status capital as $T_{i}$ and the mean status capital over n team members is $T_{mean}$, then the variation coefficient can be calculated as follows [30]:

(1) $${\left[\sum^{\;}(T_{i}-T_{mean}{)}^{2}/n\right]}^{1/2}/T_{mean}$$

Decisional capital diversity. Considering the frequency and distribution of doctors’ exchange interactions (the frequency of online interactions reflects a doctor’s capability to deal with more work under limited time constraints and exchange distribution refers to the quantity of a doctor’s decisional capital exchange behavior based on two functions, including medical articles and online consultations), decisional capital was measured by the doctors’ dynamic interactions with patients in the online consultation platform as follows: Number of medical articles that are posted by doctors on the Good Doctor platform, number of online consultations, and doctors’ online frequency [47]. Similar to the calculation of status capital diversity, we aggregated the three variables into decisional capital and then calculated the diversity using the variation coefficient.

Online reputation diversity. We measured online reputation by the total number of letters of thanks and virtual gifts, which are virtual items given by patients to doctors on the Good Doctor platform. Similar to the calculation of status capital diversity and decisional capital diversity, we averaged these two variables after normalization. Then, we computed online reputation diversity using the variation coefficient of the online reputation of all team members.

Professional knowledge diversity. Doctors in different departments often master different professional knowledge. Because team members’ departments are categorical data, we measured professional knowledge diversity using Blau’s index. First, we counted the number of team members in each department. Then, Blau’s index was calculated as follows [30]:

(2) $$1-\sum^{\;}P_{i}{}^{2}$$

$P_{i}$ in this case represents the percentage of team members in a department, and i is the number of different departments represented in the team. The index varies from 0 to a theoretical maximum of 1. A team with all members from only one department would be entirely homogenous and would hence have a diversity index of 0.

#### 4.2.3. Control Variables

At the team level, how long the online doctor team has been established may affect the team’s experience. Thus, team longevity is measured by the number of days between the day the team was established and the day we collected the data, and it was controlled for. In addition, because team scale may be related to both diversity and team performance, team size, calculated as the number of team members, was added as the control variable. Team price (Ln) is the natural log of the price of the consulting services provided by the team for patients, which may affect the number of team consultation orders. The team response rate indicates the 24-h response efficiency of a team and was added to control for team communication efficiency. Furthermore, to control for the team members’ individual performance, team average level (Ln) was also taken into account by the natural log of the average individual performance of team members. Leader reputation is the moderator and is measured by the team leaders’ online reputation. We measured leader reputation by the total number of letters of thanks and virtual gifts. Then, we averaged these two variables after normalization to compute leader reputation.

The correlation analysis among the variables was examined to help identify redundancy issues. The results show that there are no multicollinearity issues (see details in Table 2). The correlation coefficients between status capital diversity, decisional capital diversity, online reputation diversity, and professional knowledge are both less than 0.35, which shows that they are four independent variables. 

This article uses multiple linear regression models to test hypotheses. Table 3 presents the results of the hierarchical multiple linear regression analyses predicting the team performance of online doctor teams. A consultation with an expert from Shanghai No.6 People Hospital was conducted to help us explain and validate the results. We thank for her valuable suggestions in helping us explain and discuss the results. 

We entered the control variables only in Step 1. The results show that team longevity, team size, team average level (Ln), and leader reputation are significantly positively correlated with team performance (Ln). Step 1 explains 32% of the variation of the dependent variable.

Step 2 adds the main effects of the four diversity variables. The results show that status capital diversity and decisional capital diversity are negatively correlated with the dependent variable (β = −0.776, *p* < 0.05; β = −0.679, *p* < 0.001), indicating that the greater the difference in status capital and decisional capital between the team members is, the fewer consultation orders the team will have; H1 and H2 are both supported. These observations conform to the social categorization view. Online reputation diversity and professional knowledge diversity are significantly positively correlated with team performance (β = 0.249, *p* < 0.01; β = 0.292, *p* < 0.05), indicating that H4 is confirmed and in line with the information processing view. Surprisingly, H3 is not supported. Consultation from the expert indicates that this may because most doctors did not take online word of mouth as cues to classify themselves. Online reputation reflects patients’ trust and satisfaction with doctors but not necessarily the real quality of doctors, especially for doctors who have just entered the platform and have not established an initial level of trust with the patients. Meanwhile, doctors with high online reputations are often more popular, and when serving in high online reputation diversity teams, they can stimulate their peers’ enthusiasm [66] and further benefit team performance. Step 2 explains 36.1% of the variation of the dependent variable. Compared with Step 1, the addition of the main effect improves the explanatory ability of the model.

**Figure 2:**
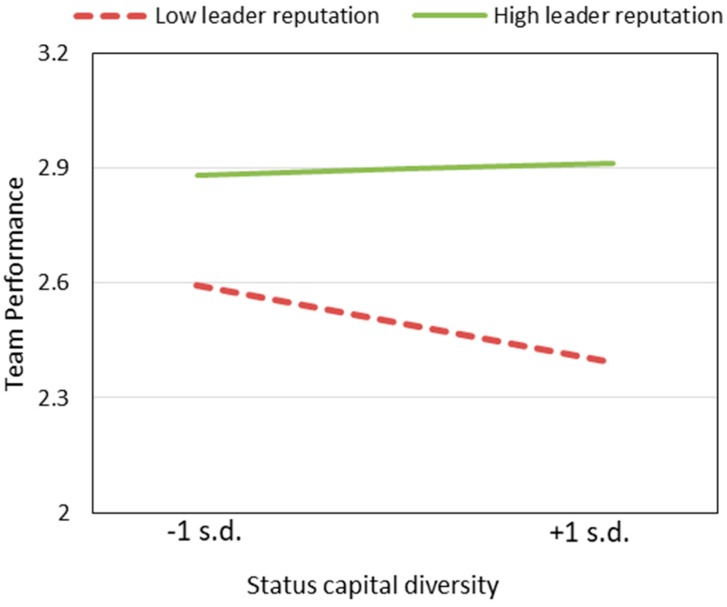
Leader reputation positively moderates the impact of status capital diversity on team performance.

**Figure 3:**
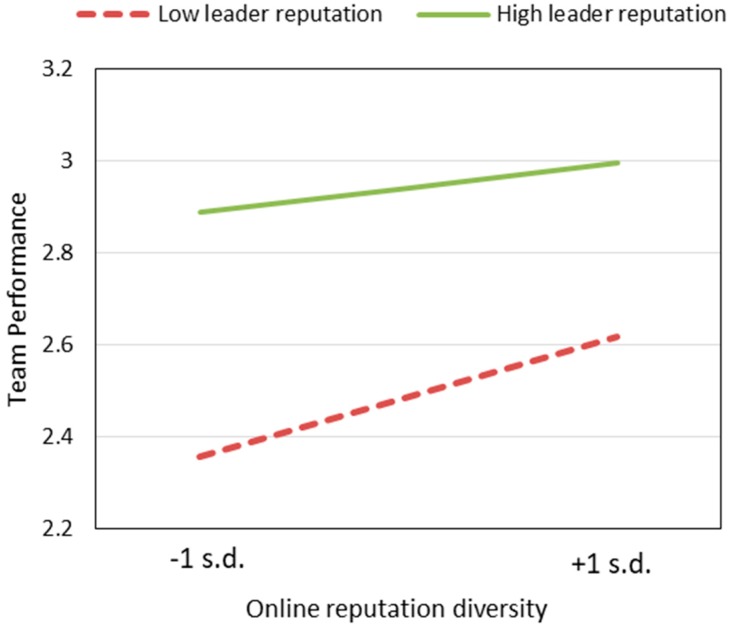
Leader reputation negatively moderates the impact of online reputation diversity on team performance.

The respective interactions of leader reputation with the four diversity dimensions are introduced into the regression equation in Step 3, Step 4, Step 5, and Step 6. The regression coefficients for the interactions of leader reputation with diversity of status capital (β = 10.22, *p* < 0.05) and online reputation (β = −1.264, *p* < 0.05) are significant. Figure 2 and Figure 3 illustrate these relationships. Leader reputation positively moderates the impact of status capital diversity on team performance (supporting H5a), indicating that the negative impact of status capital diversity on team performance will increase when the leader’s reputation is low. One possible explanation is that when there is no absolute authority in the team (leader’s reputation is relatively low), conflicts among team members will intensify and further decrease team performance. The results also indicate that leader reputation negatively moderates the positive impact of online reputation diversity on team performance (H5c is not supported), which means that the positive impact of online reputation diversity on team performance is diminished with an increase in leadership reputation. Because a team with a high reputation leader is more likely to win the trust of patients, the influence of the leader’s reputation on performance is more important than online reputation diversity. Step 3 explains 36.3% of the variation of the dependent variable. Step 5 explains 36.2% of the variation of the dependent variable.

**Figure 4:**
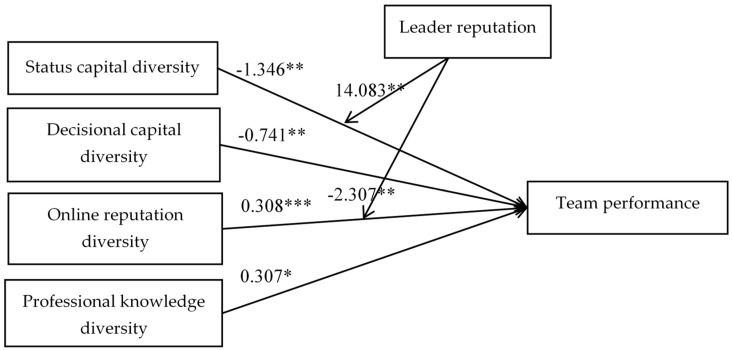
The results of four diversity dimensions and leader reputation’s moderation effects on team performance.

All the control variables, main effects, and moderation effects were introduced into the regression in Step 7. The results were consistent with previous steps. Meanwhile, R^2^ increases to 36.7%, indicating that it explains 36.7% of the variation of the dependent variable. This result shows that the final full model can best model team acquisition and leader reputations’ moderation effect on team performance. In sum, status capital diversity and decisional capital diversity are negatively correlated with the dependent variable; online reputation diversity and professional knowledge diversity are significantly positively correlated with team performance. Leader reputation positively moderates the impact of status capital diversity on team performance, and negatively moderates the positive impact of online reputation diversity on team performance (the results were summarized in Figure 4).

## 5. Conclusions

The main contribution of this research is that it distinguishes the impact of different types of diversity on the performance of online doctor teams and provides a new perspective for a comprehensive understanding of the relationship between doctor team diversity and team performance. We explore the role of leader reputation in moderating diversities and provide suggestions for team structure given different levels of leader reputation. The results showed that (1) status capital diversity and decisional capital diversity negatively affect team performance, (2) team online reputation diversity and professional knowledge diversity positively affect team performance, and (3) leader reputation positively moderates the effect of status capital diversity on team performance and negatively moderates the effect of online reputation diversity on team performance.

Our research has numerous practical implications. (1) It offers management suggestions on how to form a high-performance doctor team. For doctors who want to create or join an online doctor team, it is a good strategy to find peers with low status capital diversity and low decision capital diversity to decrease team conflicts and strengthen team cohesion. It would also be beneficial to join a team with high online reputation diversity and knowledge diversity since the former would benefit the patient’s trust in the overall team and further enhance team performance; the latter would encourage knowledge integration within groups and help avoid medical error, thus improving doctors’ service quality. Meanwhile, joining a team with high leader reputation would also be beneficial in including more consultations. (2) Our research also provides advice for the future development of online doctor teams. Strengthening medical cooperation is an important way to enhance the effectiveness of medical work [67]. Team performance is often influenced by factors such as the combination of team members’ knowledge and abilities and by the interactions among members. Positive cooperation and interaction enhance members’ confidence in team performance. The essence of online health care is to encourage doctors to actively participate while simultaneously providing patients with better services. (3) The results of our research can also provide guidance for patients choosing doctor teams on the medical platform.

The current study also has several limitations that should be addressed in future research. First, this study only uses one variable—the number of team consultations—to indicate team performance; future studies should consider other possible indicators from quality performance facet to enrich the underline mechanisms. Second, this study controls the longevity of the teams, but team establishment and development are long-term activities. Future studies should further investigate the dynamic influence of longevity factors on team performance and produce more rigorous results.

## Figures and Tables

**Table 1:** Data description and variable mapping.

Variable Type	Variable Name	Original Data	Description
Dependent Variable	Team performance	The number of team consultations	Number of team’s helping patients
Control Variables	Team longevity	Team establishment time	The date teams were established
	Team price	Team prices	Price of team consultation
	Team response rate	Team response rates	Probability of team reply within 24 h
	Team size	Team size	Number of team’s doctors
	Team average level	Members’ average number of consultations	Average individual performance of team members
Independent Variables	Status capital diversity	Hospital	Doctor’s hospital
		City	Doctor’s city
		Title	Doctor’s title
	Professional knowledge diversity	Department	Doctor’s department
	Online reputation diversity	The number of letters of thanks	Letters of thanks gave to doctor from the patients
		The number of virtual gifts	Virtual gifts gave to doctor from the patients
	Decisional capital diversity	Medical articles	Articles written by doctors in website
		Doctor’s online frequency	Doctor’s online time
		The number of consultations	Number of doctor’s helping patients
Moderation	Leader reputation	Leader’s gifts and letters	Letters of thanks and virtual gifts given to the leader from the patients

**Table 2:** Means, standard deviations, and correlations.

	Mean	SD	1	2	3	4	5	6	7	8	9	10
1. Team performance	2.634	1.433										
2. Team longevity	7.63	3.357	0.366 **									
3. Team size	3.5	1.492	0.222 **	0.184 **								
4. Team price (Ln)	3.831	1.015	0.270 *	0.117 **	0.136 **							
5. Team response rate	0.875	0.214	0.069 **	0.019	0	0.075 **						
6. Team average level (Ln)	5.953	1.656	0.429 **	0.230 **	0.039	0.273 **	0.169 **					
7. Leader reputation	0.049	0.09	0.393 **	0.107 **	0.144 **	0.386* *	0.136 **	0.504 **				
8. Status capital diversity	0.13	0.081	−0.056 *	−0.001	0.032	0.042	−0.003	−0.054 *	−0.006			
9. Decisional capital diversity	0.563	0.478	−0.268 **	0.050 *	0.172 **	−0.134 **	−0.063 *	−0.351 **	−0.200 **	0.098 **		
10. Online reputation diversity	1.066	0.446	0.98 **	−0.024	0.516 **	0.097 **	0.037	−0.015	0.219 **	0.138 **	0.342 **	
11. Professional knowledge diversity	0.19	0.261	0.168 **	0.055 *	0.099 **	0.172 **	0.071 **	0.223 **	0.196 **	0.111 **	−0.036	0.106 **

Note. N = 1568 online doctor teams; * *p* < 0.05, ** *p* < 0.01, *** *p* < 0.001.

**Table 3:** Results of regression analyses.

Variable	Step 1	Step 2	Step 3	Step 4	Step 5	Step 6	Step 7
Constant	−0.222	0.392	0.468 *	0.44 *	0.348	0.393	0.5 *
	(−1.109)	(1.83)	(2.159)	(2.016)	(1.612)	(1.833)	(2.259)
Control							
Team longevity	0.11 ***	0.125 ***	0.125 ***	0.125 ***	0.125 ***	0.125 ***	0.125 ***
	(11.856)	(13.506)	(13.548)	(13.521)	(13.509)	(13.49)	(13.578)
Team size	0.125 ***	0.124 ***	0.123 ***	0.124 ***	0.129 ***	0.124 ***	0.133 ***
	(6.037)	(5.235)	(5.231)	(5.243)	(5.403)	(5.236)	(5.596)
Team price (Ln)	0.051	0.019	0.018	0.017	0.018	0.019	0.011
	(1.512)	(0.591)	(0.542)	(0.514)	(0.562)	(0.583)	(0.323)
Team response rate	−0.056	−0.078	−0.078	−0.077	−0.075	−0.078	−0.071
	(−0.395)	(−0.567)	(−0.569)	(−0.562)	(−0.541)	(−0.567)	(−0.519)
Team average level (Ln)	0.213 ***	0.146 ***	0.146 ***	0.144 ***	0.141 ***	0.146 ***	0.13 ***
	(9.589)	(6.447)	(6.456)	(6.305)	(6.112)	(6.359)	(5.548)
Leader reputation	3.324 ***	2.883 ***	1.577 *	2.469 ***	4.619 ***	2.951 ***	3.801 **
	(8.242)	(7.126)	(2.182)	(4.536)	(3.918)	(5.005)	(3.099)
Main effects							
Status capital diversity		−0.776 *	−1.23 **	−0.786 *	−0.742 *	−0.774 *	−1.346 **
		(−2.125)	(−2.929)	(−2.151)	(−2.027)	(−2.116)	(−3.189)
Decisional capital diversity		−0.679 ***	−0.677 ***	−0.715 ***	−0.683 ***	−0.679 ***	−0.741 ***
		(−9.459)	(−9.443)	(−9.114)	(−9.513)	(−9.438)	(−9.348)
Online reputation diversity		0.249 **	0.239 **	0.243 **	0.295 **	0.248 **	0.308 ***
		(2.904)	(2.795)	(2.83)	(3.257)	(2.892)	(3.389)
Professional knowledge diversity		0.292 *	0.273 *	0.285 *	0.301 **	0.303 *	0.307 *
		(2.509)	(2.341)	(2.445)	(2.587)	(2.239)	(2.274)
Interactions							
Leader reputation * Status capital diversity			10.22 *				14.083 **
			(2.182)				(2.832)
Leader reputation * Decisional capital diversity				1.233			2.009
				(1.137)			(1.774)
Leader reputation * Online reputation diversity					−1.264 *		−2.307 **
					(−2.016)		(−2.62)
Leader reputation * Professional knowledge diversity						−0.18	−0.6
						(−0.158)	(−0.508)
R^2^	0.32	0.361	0.363	0.362	0.362	0.361	0.367
F	122.452 ***	87.976 ***	80.604 ***	80.111 ***	80.277 ***	79.930 ***	64.165 ***

Note. * *p* < 0.05, ** *p* < 0.01, *** *p* < 0.001.
